# Design of a Trichromatic Cone Array

**DOI:** 10.1371/journal.pcbi.1000677

**Published:** 2010-02-12

**Authors:** Patrick Garrigan, Charles P. Ratliff, Jennifer M. Klein, Peter Sterling, David H. Brainard, Vijay Balasubramanian

**Affiliations:** 1 Department of Psychology, Saint Joseph's University, Philadelphia, Pennsylvania, United States of America; 2 Department of Physics and Astronomy, University of Pennsylvania, Philadelphia, Pennsylvania United States of America; 3 Department of Psychology, University of Pennsylvania, Philadelphia, Pennsylvania, United States of America; 4 Department of Neuroscience, University of Pennsylvania, Philadelphia, Pennsylvania, United States of America; 5 Department of Opthalmology, Northwestern University, Chicago, Illinois, United States of America; Gatsby Computational Neuroscience Unit, United Kingdom

## Abstract

Cones with peak sensitivity to light at long (L), medium (M) and short (S) wavelengths are unequal in number on the human retina: S cones are rare (<10%) while increasing in fraction from center to periphery, and the L/M cone proportions are highly variable between individuals. What optical properties of the eye, and statistical properties of natural scenes, might drive this organization? We found that the spatial-chromatic structure of natural scenes was largely symmetric between the L, M and S sensitivity bands. Given this symmetry, short wavelength attenuation by ocular media gave L/M cones a modest signal-to-noise advantage, which was amplified, especially in the denser central retina, by long-wavelength accommodation of the lens. Meanwhile, total information represented by the cone mosaic remained relatively insensitive to L/M proportions. Thus, the observed cone array design along with a long-wavelength accommodated lens provides a selective advantage: it is maximally informative.

## Author Summary

Human color perception arises by comparing the signals from cones with peak sensitivities, at long (L), medium (M) and short (S) wavelengths. In dichromats, a characteristic distribution of S and M cones supports blue-yellow color vision: a few S and mostly M. When L cones are added, allowing red-green color vision, the S proportion remains low, increasing slowly with increasing retinal eccentricity, but the L/M proportion can vary 5-fold without affecting red-green color perception. We offer a unified explanation of these striking facts. First, we find that the spatial-chromatic statistics of natural scenes are largely symmetric between the L, M and S sensitivity bands. Thus, attenuation of blue light in the optical media, and chromatic aberration after long-wavelength accommodation of the lens, can give L/M cones an advantage. Quantitatively, information transmission by the cone array is maximized when the S proportion is low but increasing slowly with retinal eccentricity, accompanied by a lens accommodated to red light. After including blur by the lens, the optimum depends weakly on the red/green ratio, allowing large variations without loss of function. This explains the basic layout of the cone mosaic: for the resources invested, the organization maximizes information.

## Introduction

Human perception of color comes from comparing the signals from cones with different peak sensitivities at long (L), medium (M), and short (S) wavelengths. While three cone types are required to support trichromatic color vision, the three types distribute unequally. The central human fovea contains only ∼1.5% S cones, with the fraction increasing to ∼7% at greater retinal eccentricities [1]. In most dichromatic mammals, S cones are similarly rare (e.g., [2]–[4], but see [5] for exceptions). Meanwhile, the mean ratio of L cones to M cones varies widely between primate species (majority L in humans, majority M in baboons [6]–[11]). Amongst human individuals, the L∶M ratio varies between 1∶4 and 15∶1 without loss of normal color vision [12] and similar variation is seen in New World monkeys [6]. We asked: why are S cones rare, why can the L/M ratio be so variable, and why does the S cone fraction increase with retinal eccentricity?

A possible explanation for the rarity of S cones has been proposed: the lens is accommodated to focus long-wavelength (red) light; thus short wavelengths are blurred and the Nyquist limit on sampling predicts fewer S cones [13]. While plausible, this explanation seems incomplete for three reasons: (i) In human, the blur radius for blue light is ∼1.5 times the blur radius for red light ([14],[15],[16], see Results), giving, via a Nyquist sampling argument, an (L+M)/S ratio of ∼(1.5)^2^∼2; this implies ∼33% blue cones which is 5–10 times too high, (ii) The accommodation wavelength of the eye (the wavelength at which light is most sharply focused) is under behavioral control [17], and aberration could be minimized for blue light, reversing the sampling argument, if this improved vision, (iii) The sampling argument ignores noise and correlations and thus could be entirely wrong if natural scenes filtered through the ocular media had low power or greater spatial correlations at long wavelengths. To model noise and correlations, we must consider additional key factors – optical properties of the ocular media, and correlated chromatic structure in natural scenes. Thus, we asked if these two factors, in combination with chromatic aberration in the lens, might suffice to explain long wavelength accommodation and the structure of the cone array.

Our analysis treated as fixed three characteristics of the eye: (i) number of cone types; (ii) cone spectral sensitivities; and (iii) transmittance of the ocular media. Previous work has suggested that these characteristics are evolutionary adaptations to the structure of the environment. Specifically, researchers have considered the relation between the number of cone types in the retina, the spectral sensitivities of these cones, how the cone signals are processed by ganglion cells, and the statistical structure of naturally occurring spectra [18]–[20]. Researchers have suggested that the peak sensitivities of primate cone photopigments are optimally placed for encoding visual information in natural environments [21]–[24] or to facilitate crucial behavior under the constraints of chromatic aberration, diffraction, and input noise, particularly in dim light [25]. Finally, it is believed that the ocular media, especially the macular pigment, transmit less short wavelength light in order to protect the retina against damage from UV light [26]–[28].

**Figure 1:**
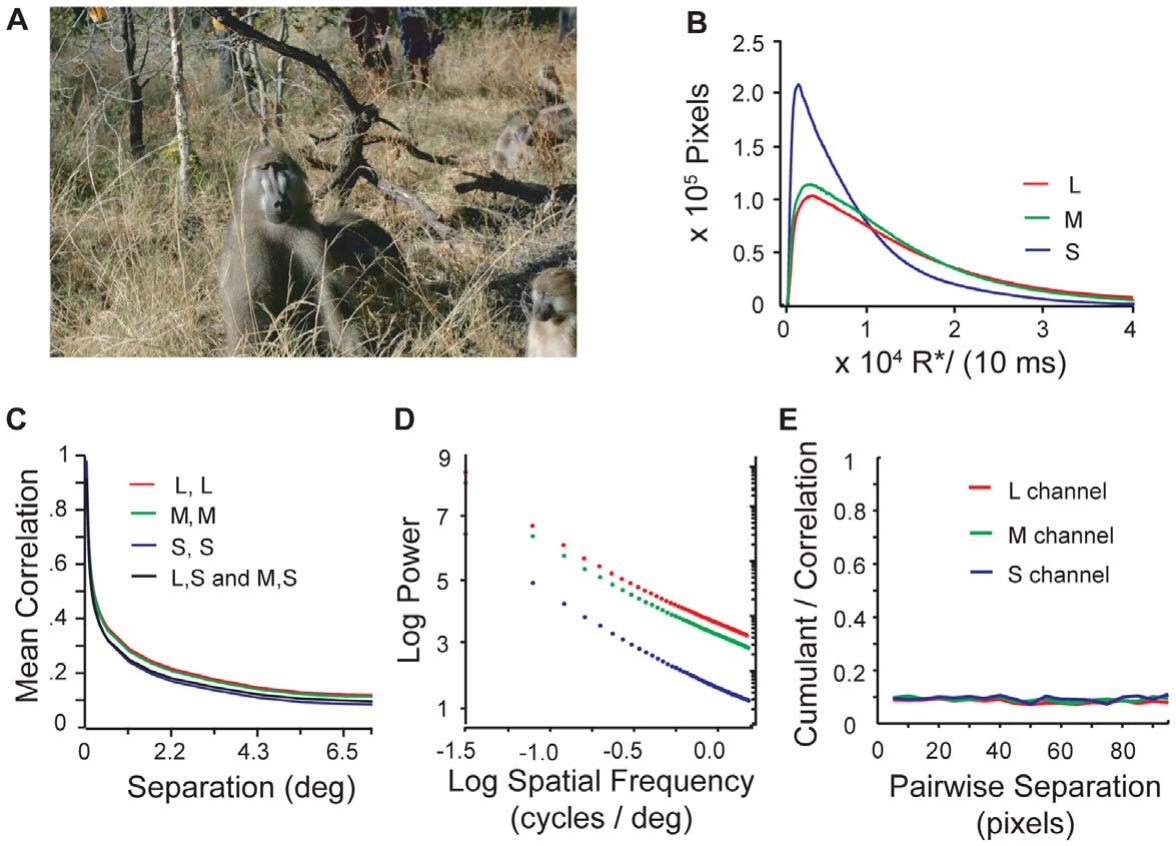
Statistics of cone responses to natural images. A) Image from Okavango Delta, Botswana image database (Photo credit: Lucia Seyfarth). B) Histogram of cone response intensity in units of cone opsin photoisomerizations per cone per 10 ms. S cone signals peak at lower values than L and M cone signals, which are similar. C) Correlation between cone signals declines with spatial separation but remain significantly positive at large separations. Correlations between cone signals from different cone types are nearly as large as the correlations between cone signals from cones of the same type. Cross-correlations between L and S cones and between M and S cones were indistinguishable and are plotted with the same black line. D) The power spectrum in each color channel shows that correlations are approximately scale invariant over several log units of spatial frequency. E) At all spatial scales 90% of the correlations between three equally separated points in an image arise from underlying pairwise correlations (see text).

To model the correlations and intensity distributions in the natural world, we based our analyses on a database of high-resolution chromatic images accumulated in a riverine savanna habitat in the Okavango Delta, Botswana (**Fig. 1a**). These images showed similar power when integrated through the spectral sensitivities of the three human cone classes. But including the selective absorption of short wavelengths by the ocular media breaks this symmetry, leading to fewer photoisomerations per second, and hence lower signal-to-noise ratio, in S cones. Measuring spatial correlations within, and between, model cone classes responding to natural images, we found surprising similarity – long-distance correlations, approximately scale invariant, prevailed between pairs of cones of any type.

Given these characteristics of cone responses to natural images, we asked, what combination of lens accommodation wavelength and cone mosaic would best support vision. Since visually guided behavior is limited by the amount of information available from the retinal cone array [29]–[31], we formulated a precise question by looking for the mosaic and lens accommodation wavelength that jointly maximized information about natural images. First, we computed the signal-to-noise ratio, or equivalently, the information rate, in single cone responses. Second, we summarized the effects of correlations in natural images by measuring how information in a cone array scaled with array size after discounting for redundancies between cones. From these data we found that in the absence of chromatic aberration, information about natural scenes was maximized by an array with ∼40% S cones and about equal numbers of L and M cones.

We then included the point-spread function of the human eye accommodated to different wavelengths [32]. The resulting blurring of light leads to redundancies in the responses of neighboring cones. Including these redundancies, information was maximized when the lens was accommodated to long wavelengths while the mosaic contained just a few percent of S cones. Meanwhile, after including optical blur, the amount of information was largely independent of the L/M ratio, allowing substantial differences in this ratio between near-optimal mosaics. In addition, plausible variations in our parameters gave a slight advantage to having a majority of either L or M cones (as seen on average in, e.g., human vs. baboon). We also modeled the topographic variation of the retina, and found that as the cone density decreased towards the periphery, the advantage of L cones decreased too. Thus, information was maximized by an S cone fraction that increased towards the periphery of the mosaic.

All of these features – low S cone fraction, large variation in L/M ratios, and peripheral increase of S cones – are seen in the primate retina. The match between our analysis and the observed structure of the cone mosaic adds to a growing body of evidence, which started with pioneering work [29],[30],[31], investigating how the constraints and properties of the biological hardware interact with the statistical properties of the natural environment to shape organization of the brain (e.g., [21], [33]–[45]).

## Results

### Image database

Our analyses are based on a new high-resolution (camera resolution: 2014×3040; 46 pixel separation = one degree of visual angle), database of color images from which we selected 176 daylight scenes of a riverine habitat in dry-season Botswana. Although our image resolution was less than that of the primate fovea, we used the scale invariance of natural images to treat the pixel spacing as being equivalent to the foveal cone spacing for a more distant observer [46],[47]. We required that the camera response be in the linear range and that fewer than 0.5% of the pixels be saturated. Most images in our database that were acquired under daylight conditions had these properties. While our images are qualitatively different from those taken in other environments (e.g., urban scenes, the van Hateren database [48], the McGill Calibrated Colour Image Database [49]), we have tested (but do not show here) that these image databases share the main statistical properties (distributions of light intensity and spatial correlations) that drive our analysis.

From the red, green, and blue camera response at all pixels in each image we estimated the equivalent L, M, and S cone photoreceptor response. First we calculated the best choice amongst linear maps between camera and cone spectral sensitivities [50] and checked the accuracy using patches from the Macbeth Color Checker imaged with both our camera and a spectral-radiometer. Photoisomerization rates (R* s^−1^) were estimated using the procedures described in [51] for guinea pig, but substituting appropriate parameters for human foveal cones (human peak photopigment sensitivities and ocular media transmittance). See Materials and Methods for details of camera calibration and image processing.

### Chromatic statistics of natural scenes

#### Distributions of photon absorption rates

Keeping fixed the number of cone types, their spectral sensitivities, and transmittance of ocular media, cone isomerization rates characterize the statistics of natural images as far as they affect the design of other parts of the system. Thus we measured the distribution of L, M, and S cone photon absorption rates for our images. These distributions were highly variable among individual images, but after averaging over images they all have a skewed shape with a low peak and long tail (**Fig. 1b**). This shape resembles the luminance distributions seen in grayscale images [46]–[48],[52],[53]. Although relatively similar raw intensities were captured by the camera within L, M and S spectral sensitivities, S cones transmit the weakest signal. This difference is primarily due to selective attenuation of short-wavelength light by the ocular media and the macular pigment [26], [27], [54]–[56].

#### Correlations among cone signals

The two-point spatial auto-correlation function (**Fig. 1c**) of signals from a particular cone type is approximately scale-invariant over several log units, leading to a power spectrum that falls off according to a power law in spatial frequency (**Fig. 1d**). This is consistent with other measured power spectra in color images [46] and grayscale images (e.g., [47]). Positive correlations persist between locations that span half the image. The cross-correlations between L, M, and S responses were nearly identical and also resembled the auto-correlations. These similarities occur despite the different spectral sensitivities of the S and L/M cones, indicating that most individual surfaces in images, even those that are perceived by humans as having vivid colors, reflect light at many wavelengths.

Natural images also have higher-order correlations among three or more points. A part of these correlations arises from underlying scale-invariant relations between pairs of points. However, since natural images contain additional object-like structures and extended contours, we asked whether there might be an additional component in the three-point correlation that is independent of the pair-wise correlations, and whether the size of this component depends on scale. The full *third-order correlation* is
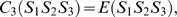
where *E* denotes the expected value of the product of signals *S_i_*, adjusted to a zero mean value. The portion of this quantity that is independent of the pair-wise correlations is given by the *third order cumulant*:
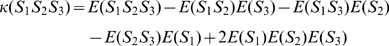
We computed the ratio κ/C_3_ for points arranged on the vertices of randomly oriented and positioned equilateral triangles of various sizes (**Fig. 1E**). The results showed that ∼10% of these three-point correlations do not derive from the underlying two-point relations and that this percentage is independent of spatial scale, in agreement with a similar analysis using the van Hateren database [57].

### Information represented by cone signals

To determine the characteristics of a cone array that maximizes information about natural scenes, we considered in turn: (**i**) the information represented by a single, independent cone signal; (**ii**) an array of cones of the same type; and (**iii**) a mixed array. The single cone analysis incorporated the attenuation of short wavelength light by the ocular media, while the cone array analyses incorporated spatial correlations in natural images. We then found the LS and LM arrays that maximized transmitted information. The optimization analysis was carried out with and without including accommodation of the lens to understand how chromatic aberration interacts with other factors.

#### Estimating information

The information transmitted by a single channel about its input depends on three factors: (**i**) the range of signals (the “bandwidth”); (**ii**) how evenly the bandwidth is used; and (**iii**) noise in the signals. Shannon captured all three of these factors in his formula for *mutual information*, which is measured by subtracting the “entropy” of noise from the “entropy” of channel responses. Taking noise to be additive, a simple approximate way of accounting for its effects is to simply bin the channel responses into levels spaced to reflect the noise amplitude. Then the mutual information between channel responses (*S*) and the input (*E*) can be estimated from the entropy of the binned responses using the formula
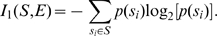
(1)Here *s_i_* represents a particular signaling level in the set of possible levels *S*, and *p(s_i_)* is the probability of *s_i_*. The range of the sum in (1) reflects the bandwidth, the probability distribution *p(s_i_)* reflects the evenness of bandwidth usage, and the spacing of levels reflects the noise. In the analysis here, *S* represents a cone's response to the image ensemble *E*. To the extent that cone signals are responding to light, their response entropy minus the entropy of noise is the mutual information between the cone response (*S*) and the image ensemble (*E*), and noise is being approximately accounted for using response bins with widths that reflect noise amplitude.

The amount of information transmitted by a signal is also related to the signal-to-noise ratio (SNR). The information capacity for a channel transmitting Gaussian distributed signals (*s*) with additive Gaussian noise (*n*) is
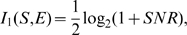
(2)where SNR is the ratio of signal power (expected value of *s^2^*) to noise power (expected value of *n^2^*). Just as in Eq. 1, large signal power (large bandwidth) increases information, and large noise for a fixed signal power decreases it. While Eq. (2) is often taken to describe a continuous Gaussian channel, this channel is effectively discretized by the noise. The scale for reliably distinguishing signaling levels is then set by the noise standard deviation. This gives the approximate connection between (1) and (2). Specifically one can separate the signal in the Gaussian channel into “distinguishable levels” *s_i_* determined by the noise standard deviations, assign each level its Gaussian probability, and then apply (1).

#### Information capacity of single cones

The photoisomerization rate (R* / cone / integration time), the first neural representation of light entering the eye, is the signal. There are two sources of noise: quantal fluctuations in photon arrival rates (photon noise) and spontaneous isomerization of cone opsins (dark noise). Thus the SNR of a given cone type is
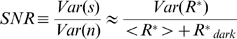
(3)where R*_dark_ is the spontaneous isomerization rate. The numerator in (3) is the signal variance and the denominator is the noise variance. To compute signal variance we measured the variance of the photoisomerization rate. Because the photon and thermal noise result from independent Poisson processes, the overall noise variance <n^2^> is the sum of the power in each kind of noise. To compute the power in photon noise, we used the fact that photon noise is Poisson. Thus, for a given light level, the noise amplitude is the square root of the signal. The power in photon noise is given by the expected value of the square of the amplitude, and is thus simply the expected value of the signal <R*>. Similarly, the power in thermal noise is R*_dark_. In the daylight conditions of our images, photon noise dominates, so we will drop the dark noise contribution entirely. S cones have, on average, fewer isomerizations primarily because of the transmittance of the ocular media (see **Fig. 1b**). Consequently, they have a lower SNR and transmit less information. Likewise, because the L and M cones have very similar distributions of isomerization rates, they will have similar SNRs and will transmit similar amounts of information.

A precise estimate of cone information rates will vary with the assumed cone integration time and the overall luminance of images in the ensemble. For a cone integration time of 10 ms, and different choices of lighting conditions for the image ensemble, the Gaussian channel approximation for single L, M and S cones gave a range of information rates ∼3 bits<*I_1L_*<∼7.5 bits, 3 bits<*I_1M_*<7.5 bits, ∼1.5 bits<*I_1S_*<∼6 bits. The broad distribution of information rates reflected a difference between scenes with direct illumination vs. shade. The estimated S cone information rate was robustly lower than the L and M cone rates, while the latter were similar regardless of the lighting conditions. Specifically, across lighting conditions the mean value of *I_1L_*–*I_1S_* was ∼1.6 bits, while the mean value of *I_1L_*–*I_1M_* was ∼0.2 bits. As we will see, this qualitative asymmetry drives the organization of the optimal cone mosaic, while the precise values of the cone information rates have little influence on the optimal cone proportions. Because visual behavior frequently requires fine discrimination in shady conditions, we analyzed the subset of shady images (typically forested and bushy scenes, which had *I_1L_*<∼5 bits). For a cone integration time of 10 ms, the Gaussian channel approximation for single L, M and S cones then gave average estimated information rates *I_1L_∼4 bits*, *I_1M_∼4* bits and *I_1S_∼3* bits. We took these estimates to mean that a cone transmitting *∼I* bits effectively has *∼2^I^* distinguishable signaling levels.

While the Gaussian channel approximation above is one way to estimate the information transmitted by a single cone, another approach is to directly apply Shannon's formula (1) to the cone isomerization distributions in **Fig. 1b**, binned to reflect photon noise. This method similarly gives less information in the S cone signals and approximate equality between L and M cones. Our main result using this alternative formulation for the single cone information is given in Materials and Methods.

Information transmitted by an array of cones of the same type. If the responses of all cones were statistically independent, the information transmitted by a cone array of a given type would simply be the number of cones in the array times the information transmitted by a single cone. However, cone signals are not independent and are correlated over long distances (**Fig. 1c**). These correlations cause redundancy in the signals of nearby cones, so that the information *I_N_* represented by an array of cones scales sub-linearly with the number of cones in the array (*N*).

Following Eqs. 11–13 in Borghuis et al. [58], the information represented by the response of an array of *N* cones about an image ensemble is taken to scale as
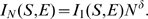
(4)where *I_1_* is the information in a single cone and 0<δ<1. This power law dependence is plausible over a large range of array sizes because the pair-wise correlations in natural scenes are approximately scale-invariant over several orders of magnitude in separation (**Fig. 1d**; [46],[47]) [58]. The pair-wise scale-invariance is usually taken to arise from the scale-invariance of images as whole [53],[59]. Cone isomerizations, unlike signals at later stages of retinal processing, are not decorrelated, and therefore have the same long-range redundancy found in the images they encode. Thus, our task was to estimate δ from the image data.

**Figure 2:**
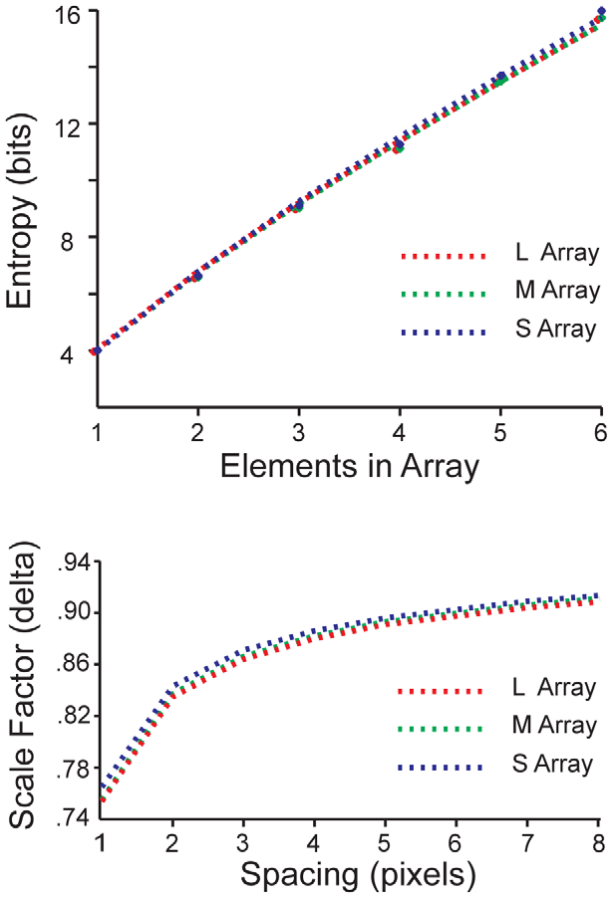
Information in arrays of L, M, and S cones. (Top) Information in *N* cones of a given type, I_N_, plotted as a function of *N* for L, M, and S cone arrays. Array spacing is set here to 1 pixel (46 pixels = one degree of visual angle). The best-fitting power law (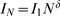) is also shown. For the minimal spacing of pixels in our images (*d* = 1), δ = 0.75 in each channel. Using 50 randomly selected images from the van Hateren database [48] similarly gave δ = 0.72. (Bottom) δ is plotted as a function of spacing (*d*) for each cone class.

We estimated δ by directly measuring how the amount of information represented scales with the size of small arrays. First we generalized Eq. (1) to an array of cones
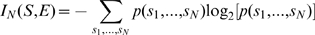
(5)where *p(s_1_,…s_N_)* is the joint probability of the *N* cone signals. The scale invariance of natural images was used to treat each pixel in an image as a model cone, with responses discretized to 16 equally probable levels. The scaling exponent δ was estimated by fitting to the measured information in small arrays (*N = 1…6*; **Fig. 2** **, top**). The data are not well-fit by a straight line (note that the errors bars on the data points are tiny because our sample is so large, allowing us to distinguish between the linear and power-law fits). Specifically, if there were no correlations the information would have grown as *N log_2_(16)* giving 24 bits for *N = 6*. Likewise, if we had only included nearest neighbor correlations, then the information in a 6-pixel array, estimated as three times the information in pixel pairs, would have been about 6% higher than measured in (**Fig. 2** **, top**). We checked that the estimated scaling exponent did not depend significantly on the number of discrete levels. In mixed cone arrays, the spacing of the cones of a particular type will depend on the proportion of the cones of that type in the array. To reflect this we calculated how δ varies for arrays with different spacings between elements (**Fig. 2** **, bottom**). For all spacings we found that δ was essentially identical for L, M, and S arrays, reflecting the very similar correlations within each of these frequency bands. Trying to estimate high dimensional entropies using (5) is difficult (see, e.g., [60]) – hence in subsequent analyses we tested to what degree variations in the estimate of δ affected our results.

### The mixed cone mosaic without chromatic aberration

Having estimated information in single cones and in arrays of one type of cone, we asked how a mosaic with two cone types should be organized to maximize information. To separate out the effects of accommodation wavelength of the lens we first studied the optimal mosaic without chromatic aberration.

The information in a mixed mosaic of two cone types is the sum of the information in each array minus the redundant mutual information between them. For example, the information transmitted by a mixed array of X and Y cones (where *X* and *Y* can be *L*, *M* or *S*) is given by
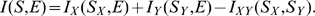
(6)Here *S_X,Y_* are the sets of X and Y array responses while *S = {S_X_,S_Y_}* represents the set of joint responses; *I_X_* and *I_Y_* represent the mutual information between the responses of the *X* and *Y* arrays and the image input; and *I_XY_* is the mutual information between responses of the X and Y subarrays:
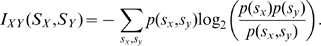
Here *p(s_x_,s_y_)* is the joint response distribution of X and Y cone arrays, and *p(s_x_)* and *p(s_y_)* are marginal response distributions of each cone type. In deriving (6) we assumed that noise in X and Y cones is uncorrelated, so that the conditional response probability factorizes: *p(s_X_,s_Y_|E) = p(s_X_|E) p(s_Y_|E)*.

Neglecting chromatic aberration, the information scaling for each cone type (see (4) and **Fig. 2**) then allows us to write
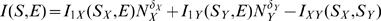
(7)Here, *I_1X_* is the information transmitted by a single X cone; *I_1Y_* is the information transmitted by a single Y cone; and δ*_X_* and δ*_Y_* are scaling exponents. The number of cones is *N = N_X_+N_Y_*, while the average distance in pixels between neighboring cones is *d_X_* = *√(N/N_X_)* for X cones, and *d_Y_ = √(N/N_Y_)* for Y cones. The exponents δ*_X,Y_* are functions of *d_X,Y_* (see **Fig. 2**).

**Figure 3:**
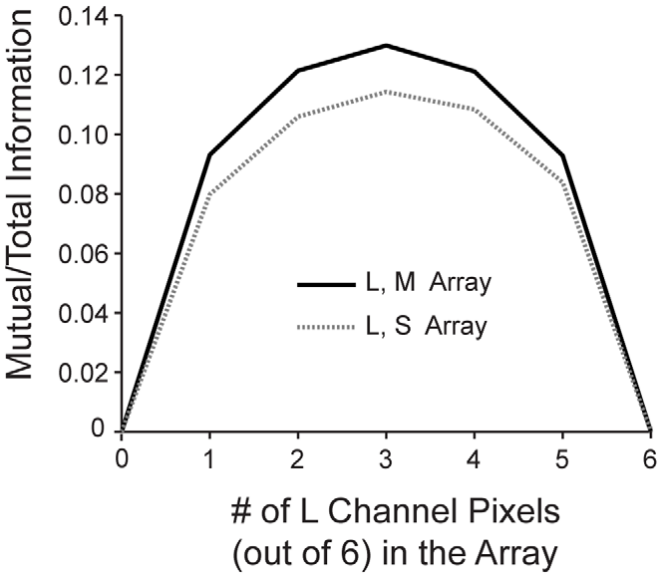
Mutual information between cone arrays. Mutual information between L pixels and M pixels (solid line) and between L pixels and S pixels (dotted line) is shown as a function of the number of L pixels (out of 6) in the array.

We measured the mutual information *I_XY_* in a 6-pixel array by varying the proportion of each kind of cone and their geometric arrangement. The cone signals were discretized to reflect the number of signaling levels in each cone class, commensurate with their different estimated information transmission rates. These discrete signals were then used to directly compute mutual information using (5). In this way, the mutual information between the L and M and between L and S cones in mixed arrays was estimated directly from the image data. To do this we used (5) to compute the total information (*I*) in mixed arrays, as well as the information in the subarrays of each type. From (6), this gave *I_XY_(S_X_,S_Y_) = I_X_(S_X_,E)+I_Y_(S_Y_,E)−I(S,E)*. We averaged over all geometric arrangements of arrays with the same cone proportion, to smooth out effects of pixelation. As expected, the mutual information between cone types vanishes for arrays with only one cone type and peaks in between, giving a domed shape (i.e., mutual information between two cone classes was highest when the array had an approximately even mix of the two classes of cones; **Fig. 3**).

The above analysis estimates how the mutual information between X and Y cones changes with the proportion of X and Y cones in a small array. Now we need to know how the mutual information in a *large* array changes with the proportion of cones of each type. This is hard to measure directly but will have the same qualitative domed form as for small arrays. Thus to extrapolate to large arrays we made the simplifying assumption that the ratio
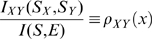
is a function only of the relative fraction of X cones, *x = N_X_/N* (or Y cones, *(1-x) = N_Y_/N*). Using this form of the mutual information between X and Y cones, we can rewrite (7) as
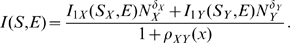
For arrays in which the scaling exponents of the two subarrays are similar (δ*_X_*≈δ*_Y_* = δ), our simplification is equivalent to assuming that total information in the array also scales as *N* ^δ^.

The similar correlations between L,M,S cones and slow variation of δ with spacing in **Fig. 2**, thus imply that our simplification should be valid for mixed arrays with roughly similar numbers of cones of each type, and for sparse arrays, since δ*_X_*≈δ*_Y_* = δ in these cases. We checked that our final results were self-consistent within this domain of validity. We also checked that our final results depended largely on the qualitative *shape* of the X-Y mutual information curve (which we infer from small arrays), rather than the precise values. We confirmed this by repeating our analyses with various assumed domed shapes for the mutual information.

**Figure 4:**
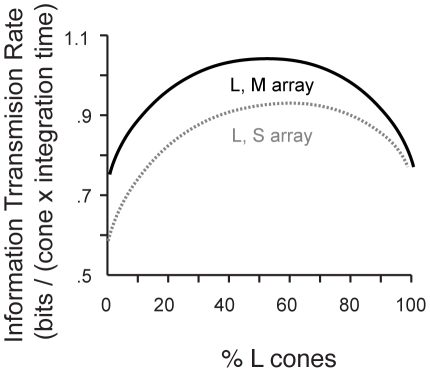
Optimal mosaic for a mixed cone array. Information transmitted by a mixed LM array as a function of the percentage of L cones in the array is shown (solid line), and similarly for an LS array (dotted line). Information represented by a mixed LM array was highest with 52% L cones. Information represented by a mixed LS array was highest with 61% L cones. These results do not include the effects of the human eye's optics.

To find the optimal mixed cone mosaics we first measured the ratio ρ directly from the result in 6-pixel LM and LS arrays (**Fig. 3**) and used our estimates of single cone SNR to write
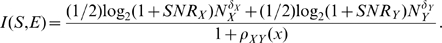
We then obtained the optimal mosaic by maximizing *I(S,E)* with respect to the proportion of L cones. We obtained an optimal LM mosaic with 52% L cones and 48% M cones. Using the same technique, but substituting S cones for M cones, we found an optimal LS mosaic with 61% L cones and 39% S cones (**Fig. 4**).

In both cases, the small excess of L cones was driven by two factors: (a) the similarity in power and correlations between L, M and S sensitivity bands in natural scenes, and (b) the selective attenuation of short wavelength light by the ocular media, which breaks the symmetry between L, M, and S. Because L and M bands are so similar, the optimum contained about equally many of each type of cone. Meanwhile, the higher SNR in L responses resulted in 20% more L cones in the optimal mosaic. This mosaic, which is well adapted to the symmetric statistics of natural images and to the attenuation of the blue light in the ocular media, differed in two respects from the observed characteristics of the human eye: (a) the S cone fraction is an order of magnitude too high, and (b) there is no indication that the L/M ratio can be any more variable that the L/S ratio without detriment. But this analysis omitted one further key factor – chromatic aberration in the lens.

### Optical blur makes S cones rare and reduces sensitivity to the L/M ratio

The lens of the eye blurs light of different wavelengths to different degrees. Such chromatic aberration can affect the cone proportions in the optimal mosaic because the amount of blurring differs for each cone channel [61]. The presence of blur modifies the problem of calculating information in an array: within a blurred region the information conveyed by pixels is highly redundant.

For each color channel the extent of the optical blur was estimated from measurements of optical aberrations in human observers ([32]; data and code provided by H. Hofer; see Materials and Methods). While the eye can accommodate to various wavelengths, in white light and under normal viewing conditions the eye tends to focus for longer wavelengths, near the peak sensitivities of L and M cones (e.g. [15],[16]). For this reason, we used the mean chromatic point spread function (PSF) averaged across 13 subjects when accommodation focuses light best on the L and M cones. PSFs are highly kurtotic, and so values far away from the central peak are important for characterizing the PSF width. As an estimate of the region over which the blur is large, we chose one half of the radius that enclosed 90% of the PSF as a measure of this width. This choice corresponds roughly to twice the full-width at half-height of the PSF. For each color channel, this estimate of the spatial extent of the significant chromatic aberrations (i.e., blur) gave
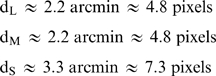
with the conversion to pixels obtained assuming a foveal cone-to cone spacing of ∼2.2 cones/arcmin [62]. To estimate the information in a blurred chromatic mosaic, we made the approximation that chromatic aberrations render L, M, and S cones separated by distances less than *d_L_*, *d_M_*, and *d_S_* respectively, completely redundant. We also made the approximation that, for each cone class, the blur has no effect beyond this distance.

First we consider cones of one class separated by distances less than *d_X_*. In our approximation such cones are transmitting completely correlated signals but have independent noise. Averaging *n* redundant signals that are each corrupted by an independent noise source (each with the same average magnitude) will increase the signal-to-noise ratio by a factor of *n*. Thus, in our analysis, averaging across a block of *n* redundant cone signals increases the signal-to-noise ratio relative to a single cone so that the block of cones represents
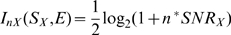
bits of information. The information represented by a block of *(d_X_)^2^* pixels is therefore
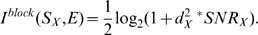
This block information can be thought of as the information represented by a single ‘effective pixel’ that includes all the redundant pixels in a small region. In mixed arrays of cones of different types, a given block may only contain a fraction *x* of cones of a particular type. Then,
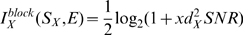
gives the information represented by pixels of that type within the block.

To compute the total information in an array of cones, we group pixels into blocks of area 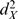 and treat the blocks as mutually correlated in a scale invariant way. Specifically, each of the blocks defined by the blur space constant is treated as a single effective pixel transmitting *I^block^* bits. Then, taking these blocks as being spatially correlated as in **Fig. 1d** (because of the scale invariance of natural scenes), the same treatment as for arrays of single cones can be applied to arrays of blocks of cones. Thus, following (4) for single cones, the total information transmitted by cones of one type in the blurred array is given by a power-law in the number of blocks:
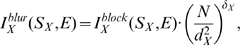
where *N* is the total number of pixels in the array, and so *N/d_X_^2^* is the number of blocks in the array and δ_X_ is the scaling exponent from **Fig. 2** (bottom) for a pixel separation equal to the spacing of the blocks. For L, M blocks spaced at 4.8 pixels, this gave δ*_L,M_ = 0.89*, while for S blocks spaced at 7.3 pixels, δ*_S_ = 0.91*.

The total information in a mixed array is then approximated as the sum of the information in the sub-arrays of each type minus the mutual information between the sub-arrays. This mutual information was taken to be the same fraction of the total information as measured before blurring. This approximation reproduced the general domed shape of the mutual information as a function of the fraction of cones of each type. Thus the information in an array with two kinds of cones and blurred optics was estimated as
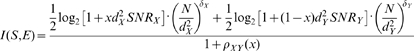
(8)where *x = N_X_/N* and *1-x = N_Y_/N = (N-N_X_)/N* are the fractions of each kind of cone. Including blur in this way increases the redundancy in each channel, predominantly among nearby pixels.

**Figure 5:**
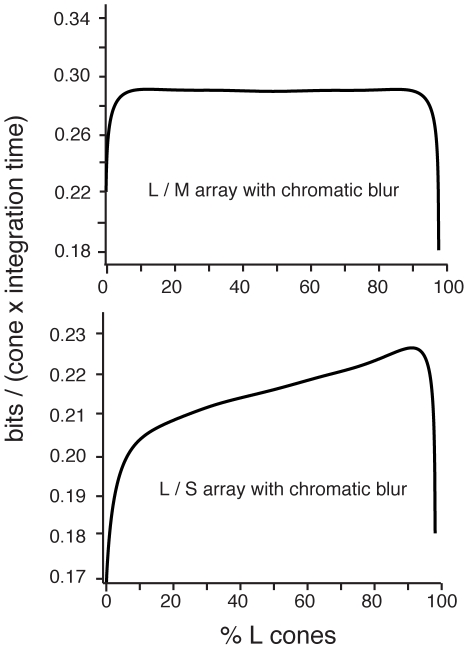
Optimal mosaic after accounting for chromatic aberration. (Top) Information represented by a mixed LM array as a function of the percentage of L cones in the array is shown on the top, and similarly for an LS array (Bottom). When we model the effects of chromatic blur due to human eye optics, information transmitted by a mixed LM array was largely independent of the L/M ratio, except when one type was extremely scarce. Information transmitted by a mixed LS array was highest with ∼6% S cones.

We asked what cone fractions maximized information when the optics are accommodated to focus light best on L and M cones. Using our estimated values for the blur (*d_L,M,S_*), the scaling exponents (δ*_L,M,S_*), the SNRs, and the redundancy (ρ*_LM_*, ρ*_LS_*) we plotted the total information conveyed by LM and LS arrays (**Fig. 5**). The increased redundancy in the L and M channels produces a broad range of equally effective LM mosaics (**Fig. 5** **, top**). That the L/M cone ratio has little effect on the information transmitted by a cone mosaic is consistent with the large variability in L/M ratios in primates with normal color vision [6],[12,] and with the observation that human performance on some psychophysical tasks is invariant with respect to cone ratio [63],[64].

Meanwhile, the blur reduces the information transmitted by the S channel more than by the L and M channels since the blur in the S channel extends further. Thus its inclusion reduces the number of S cones in the optimal mosaic as compared to **Fig. 4**. That most information is transmitted by an array with few S cones (∼6.5% - **Fig. 5** **, bottom**) is consistent with the rarity of S cones in most mammalian cone mosaics (e.g. [53]). The advantage of L cone domination is small but significant – using our parameters, a mosaic with 90% L cones conveys 10% more information per cone than a mosaic with 90% S cones. This result confirms the basic intuition of Yellott et al. [13], that chromatic aberration plays a key role in the organization of the cone mosaic, but includes additionally the effects of spatial correlations and noise.

One limitation of our analysis was that we analyzed a mixed mosaic of just L and S cones; if we were to consider all three cone types simultaneously, the fraction of S cones in the optimal mosaic would likely decrease. This is because an optimally organized LM mosaic transmits slightly more information per cone than a mosaic with only L cones. Thus an optimal trichromatic mosaic would have still fewer S cones than the optimal LS mosaic. Likewise, because the fraction of S cones in the optimal array is small, it is unlikely to significantly affect the L/M ratio in the trichromatic array.

### How variations in optical factors and scene statistics affect the optimal array

Our results for the optimal mosaic are due to the interaction of four factors: (**i**) correlations within a cone class (summarized by the scaling exponents δ*_L,M,S_*); (**ii**) correlations between cone classes (summarized by the redundancy factor ρ*_LS,LM_*); (**iii**) optical blur (summarized by the blur widths *d_L,M,S_*); (**iv**) power in different chromatic bands and attenuation by the ocular media (summarized by single cone SNRs). All of these factors were modeled and estimated, rather than directly measured, and might vary between individuals and species. Thus, to test the relative importance of each of these factors in determining the optimum we systematically varied each one while keeping the others fixed and determined the consequences for the optimal array.

#### Variations in scaling

**Figure 6:**
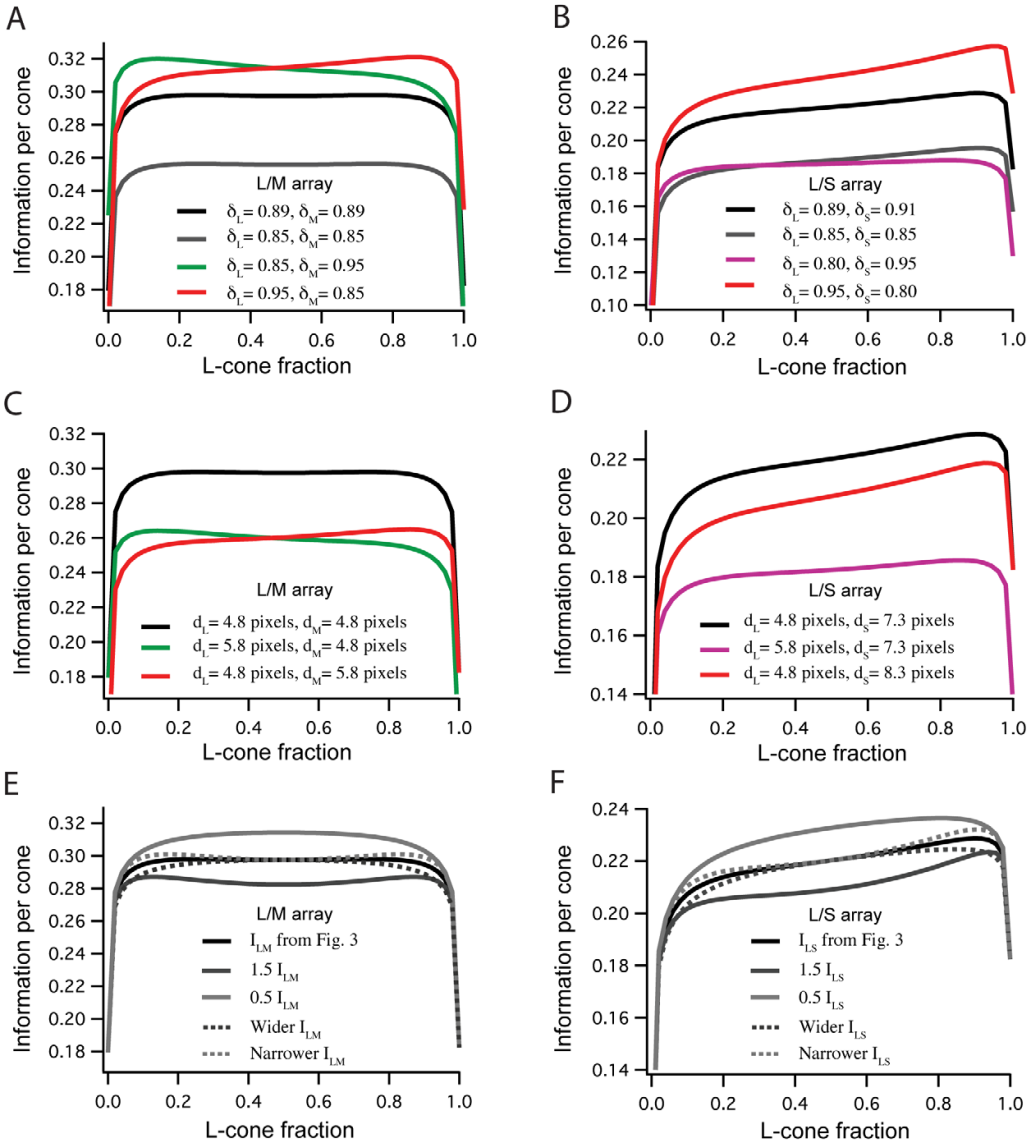
Variations in the optimal mosaic. Information per cone as a function of L cone fraction is shown for various scaling exponents, chromatic PSFs, and forms of the mutual information between cone classes. (a) Varying the scaling exponents δ*_L_* and δ*_M_* jointly had little affect on the flatness of the LM information curve. Differential scaling in L vs. M of about 10% led to approximately 3% higher information transmission rate for L or M dominant arrays (depending on which channel scaled with higher exponent). (b) Varying the scaling exponents, δ*_L_* and δ*_S_* had little affect on the optimal ratio of L and S cones, unless the L channel scaled with a substantially smaller exponent than the S channel. (c) Increasing blur in the L or M channel (while keeping the other channel's blur fixed) led to an M or L dominated optimal mosaic, respectively. A 25% increase in blur was necessary to incur a 5% advantage for a mosaic dominated (90%) by one cone class. (d) A 25% increase in the blur in the L channel relative to the S channel was necessary to significantly reduce the advantage of an L cone dominated mosaic relative to an S cone dominated mosaic. (e) Adjusting the peak or width of the form of the LM mutual information curve (see **Fig. 3**) had small effects on the flatness of the LM information curve. A more peaked or narrower mutual information (see text) curve led to a 2% advantage for either an L or M dominant mosaic. A less peaked or wider mutual information curve led to a 1% advantage for an evenly mixed LM mosaic. (f) The same adjustments to the LS mutual information curve had little effect on the optimal L/S ratio.

First we studied variations in the scaling exponents δ*_L,M,S_* that summarized an effect of scene statistics – spatial correlations within each cone array. Given the L, M, S block spacing (*d_L_ = d_M_ = 4.8* pixels, *d_S_ = 7.3* pixels), we had measured δ*_L_* = δ*_M_* = *0.89* and δ*_S_ = 0.91* from **Fig. 2**. We found that varying δ*_L,M_* jointly simply moved the flat LM information curve up or down but a 10% difference between δ*_L_* and δ*_M_* gave an 3% advantage to having 90% L or M cones (**Fig. 6a**) as opposed to 50%. For LS arrays we found that varying δ*_L_* and δ*_S_* together, while keeping them similar, simply shifted the height of the information curve (**Fig. 6b**). However, a substantial (10%) difference between δ*_L_* and δ*_S_* sharpened or reduced the advantage of L-domination in the array (15% vs. 5% more information with 90% L cones than with 90% S cones). Thus, the measured similarity in correlation within each cone sensitivity band plays a key role in the organization of the optimal mosaic.

#### Variations in blur

We estimated the region over which optical blur is large in terms of the chromatic point spread function averaged over many observers. Differences between observers or a different definition of the blur width could lead a different estimate. Thus we tested that rescaling the blur widths as *d_L,M,S_′ = c d_L,M,S_* or shifting them together as *d_L,M,S_′ = c + d_L,M,S_* affects the overall height of the information curves, but has little effect on the cone proportions in the optimal array (not shown). Then we tested the effects of relative changes in the blur (**Fig. 6c,d**). For LM arrays we found that the cone type with the smaller blur will dominate the optimal array. For LS arrays increasing the blur of L while S blur stays fixed reduced the advantage of L cones. In both cases, a 25% change in the relative blur widths was necessary for a 5% change in information per cone conveyed by an array with 90% L vs. an array with 10% L. We concluded that our results were robust to modest relative variations in the blur estimates, and that the cone fractions in the optimal mosaic have similar sensitivity to variations in optical blur as compared to variations in the spatial correlations (**Fig. 6a,b**).

#### Variations in mutual information estimate

We extrapolated the mutual information between cone classes from small arrays (**Fig. 3**) by assuming that redundancy within an array is only a function of cone fraction. To test the dependence of our results on the exact form of the mutual information, we parameterized domed shapes similar to those in **Fig. 3** as
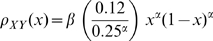
Here β fixes the overall normalization while a larger α parameterizes a narrower curve. The choice α = *0.7*, β = *1* matches the LM curve in **Fig. 3**. Variations in the width of ρ*_LM_* had only small effects on the LM information curve – between α = *0.4* (wider ρ) and α = *1* (narrower ρ), the LM information curve remained very flat (**Fig. 6e**). However, larger α gave a small advantage to having a majority of L/M cones as opposed a 50/50 balance (α = *1* gave a 1% advantage). Smaller α, meanwhile, made a 50/50 balance slightly advantageous. Varying the overall amplitude of ρ*_LM_* had similar small effects – a 50% increase in β gave a 2% advantage to having a majority of L/M cones, as opposed to a 50/50 balance. Thus, over a wide range of parameters the LM information curve is quite flat, but a modest advantage can develop for having majority L or M. Finally, variations in ρ*_LS_* made little difference to the cone proportions in the optimal LS array (**Fig. 6f**).

#### Variations in SNR

We investigated how the estimates of single cone SNR affected the optimal cone proportions. Surprisingly, the optimal proportions depended weakly on these parameters so long as they were large – e.g. a 5–10-fold increase in the estimated SNRs increased the optimal L cone fraction by ∼3–4%. To understand this, we observed that when the SNRs are large, and *x* (the fraction of cones of type X) is not too close to 0 or 1, the condition for the optimum (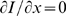) turns out to be
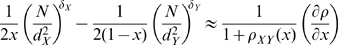
Thus, to leading order, the cone SNRs drop out of the balance condition determining the cone proportions in the optimal array when chromatic aberration is included. In the absence of blur, the lower SNR of S cones by itself resulted in an optimal array that contained fewer (∼40%) S cones (Fig. 4). However, the inclusion of optical blur further reduces the optimal S cone proportions, and, given that the symmetry between L and S cones is already broken by the lower S cone SNR, the optimal proportions are determined to leading order by spatial correlations and optical blur.

#### Summary

The solution for the optimal cone array is driven by a balance between three factors – the blur as summarized in *d_X,Y_*, the spatial correlations within cone classes as summarized in the scaling exponents δ*_X,Y_*, and correlations between cone classes as summarized by the redundancy factor ρ*_XY_*. While the LM information curve remains relatively flat over substantial range of these parameters, a small advantage can arise for having a majority of L or M cones, or for having a 50–50 array. Possibly this explains why the mean L-fraction in different species seems to be skewed towards L (human, [12]) or M (baboon, [11]), while the average across primate species may be close to an even (50–50 LM) mix [6]. At the same time the relative flatness of the LM information curve across a wide range of parameters likely explains why large variations in L cone proportion apparently occur across individuals without impairment of vision [63],[64]. Meanwhile, across a broad array of variations the optimal LS array robustly had a majority of L cones, although the information advantage of this organization varied somewhat with the parameters. We found that spatial correlations in the cone array, which arise from natural scene statistics, were as important in determining the optimum as optical blur, which arises from a property of the lens.

### Effects of accommodation

The analysis above was carried out with a lens that focused long wavelengths best, as appropriate for the normal accommodative state of the eye [15],[16]. However, the accommodation wavelength at which light is most focused by the lens is under behavioral control. Since the single cone SNRs, provided they were large, were a sub-leading determinant of the optimal cone fractions, we wondered whether there is any advantage to long-wavelength accommodation.

Thus we explored how different accommodation wavelengths affect the distribution of cones in the optimal LS mosaic. Using the polychromatic PSFs computed for various accommodation wavelengths (see Materials and Methods), we repeated the optimization procedure (described above) for mixed LS arrays. Since long and short wavelengths cannot be focused simultaneously, we expected to find optimal arrays that are dominated by either L or S cones, depending which channel is best focused.

**Figure 7:**
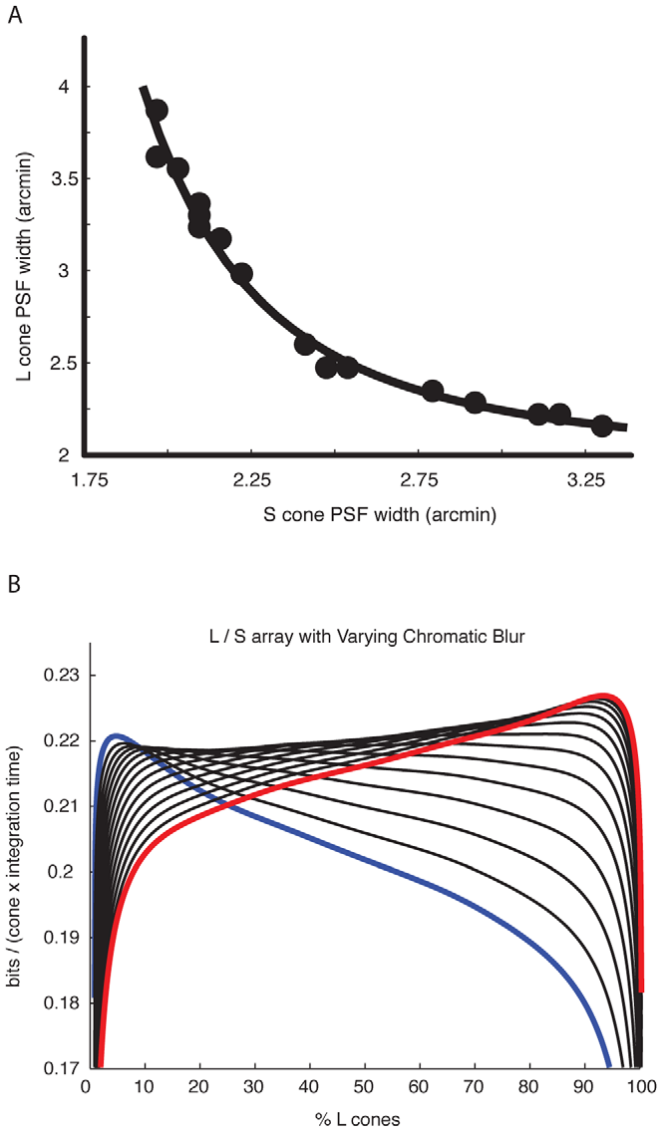
S channel vs. L channel chromatic aberration. (a) As the eye accommodates, the S cone and L cone PSFs trade off. The PSF can be decreased in width for one cone class at the cost of increasing its width for the other. Widths shown are one half of the radius that enclosed 90% of the PSF. (b) The LS information curve is shown for varying accommodation. When the lens focuses shorter wavelengths, the optimal mosaic favors S cones (blue line). When the lens focuses longer wavelengths, the optimal mosaic favors L cones (red line).

Our measure of the spatial extent of the blur in each color channel was again half of the 90% width of the PSF. The width of the L cone PSF is plotted against the width of the S cone PSF in **Fig. 7a**. For each accommodation wavelength, we then estimated the information per cone as a function of the L cone fraction following Eq. 7 (**Fig. 7b**). When the lens accommodated to the L cone peak sensitivity, the mosaic maximizing information per cone had mostly L cones, while a lens accommodated to S cone peak sensitivity led to an optimal mosaic with mostly S cones.

**Figure 8:**
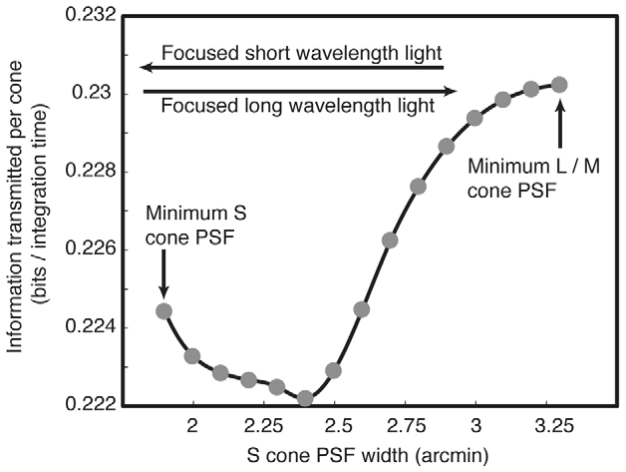
Effects of accommodation on information transmission rate. The information transmitted per cone is highest for an optimal arrangement of L and S cones when light is focused for L cone signals, and consequently more blurred for S cone signals. Results are shown for an array of N = 1000 cones; similar curves result for *N* between 10^3^ and 10^6^.

The information per cone in the optimal mosaic for each accommodation wavelength (parameterized as S cone PSF width) is plotted in (**Fig. 8**). Information transmission rates were highest when L cone light was focused sharply and S cone light was blurred. Although the per cone advantage of focusing long wavelength light is small (∼3%), multiplying by the number of cones in a retina gives a significant increase in the total amount of transmitted information. Interestingly, the worst choice is to accommodate *between* the L and S peak sensitivities. Focusing short wavelength light could be advantageous if, due to some other constraint, it was impossible to focus long wavelength light sufficiently. In this case, the optimal retina has mostly S cones, which may be related to the existence of a few species with S cone dominated retinas [5].

One might wonder whether the apparently small excess of information (∼3%) in the optimal accommodation wavelength in **Fig. 8** actually confers a significant selective advantage. It is worth noting that small selective advantages have a multiplicative effect over generations, and, just like compound interest, can pay large dividends over evolutionary time.

### The optimal cone mosaic varies with eccentricity

**Figure 9:**
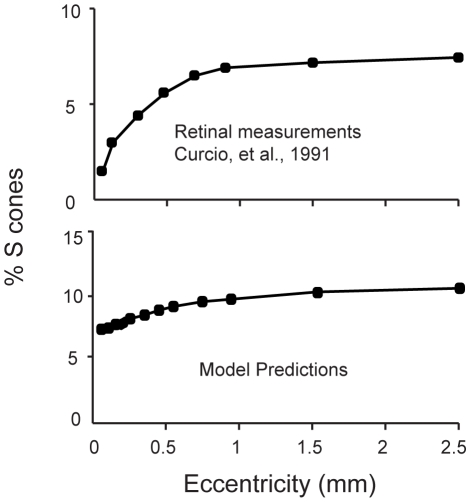
Optimal mosaic as a function of retinal eccentricity. (Top) Variation of measured S cone proportion with retinal eccentricity in human [1]. (Bottom) Proportion of S cones increases with eccentricity in the optimal array.

Our analysis thus far has considered the overall proportions of cones of different classes. An additional observation is that the fraction of S cones in the human retina increases somewhat with eccentricity [1],[65],[66]. We wondered whether this observation might also be accounted for by our theory. Two relevant factors that are known to *decrease* with eccentricity are the overall cone density of the mosaic [1] and the optical density of short-wavelength filtering macular pigment [50]. The effect of reducing macular pigment density will be to reduce the SNR advantage of the L and M cones over the S cones, and to the extent this has an effect this would tend to increase the relative proportion of S cones. Our analysis of robustness presented above, however, indicates that this effect will be small but in the right direction, and preliminary calculations (not presented here) indicated that alone it would be insufficient to account for the increase in ∼1.5% to ∼7% S cone percentage from the central fovea to the periphery. We thus focused on the effect of the decrease in overall cone density. As the distance between cones becomes large relative to the blur, the number of cones in each blurred and redundant block decreases, reducing the significance of the blur in the optimization. Since chromatic aberration has a greater effect on the S-channel, increased sparseness of the array tends to increase the fraction of S cones in the optimal mosaic. To see this, we kept the extent of the blur fixed, and used the scale invariance of natural images to treat the image pixels as having the separation of cones at larger eccentricities that are viewing the same scene from a greater distance. Repeating the analysis for the optimal mosaic, we found that the predicted S cone fraction increases with decreasing cone density (**Fig. 9**). Overall the predicted optimal cone fractions are somewhat higher than seen in Curcio et al. [1], but these measurements were accumulated from only two retinas and variations should be expected between individuals. Moreover, our estimates of the exact optical parameters to use for the periphery are not currently precise enough to support inferences about the significance of predicted differences of a few percent. The key point we wish to emphasize at this juncture is thus that our theory is qualitatively consistent with an increase of S cone proportion with eccentricity.

### Robustness of results

The results presented here depend on many estimated and modeled quantities and thus it was important to test how plausible variations in these quantities might affect the optimal mosaic. First, we checked that our results were insensitive to the details of the model of the eye's optics, and confirmed that essentially the same results were obtained when we used the Marimont and Wandell [61] model of the eye's chromatic aberrations.

We also approximated the cone signal as a Gaussian channel so that an explicit functional form for information could be manipulated. As a check, we directly estimated the information in the cone signal from the histogram of isomerizations rates binned according to the Poisson noise at each rate. This procedure gave a similar result to the Gaussian approximation – our results follow from the similarity of SNR in the L and M cone channels, and the smaller SNR in the S cone channel. The relative sizes of SNR in each channel are a consequence of the cone spectral sensitivities (which peak at similar wavelength for L and M cones, but at significantly shorter wavelength for S cones) and the transmittance properties of the ocular media, which selectively attenuate short wavelengths.

To extrapolate information to large arrays we used the power law that gave a good fit for small arrays. We checked that the results were insensitive to the overall size of the array by varying the number of model cones (*N* in (4)) between 1000 and 1,000,000. In these extrapolations we treated noise in photoreceptors as being dominated by photon noise and therefore independent. It should be kept in mind that noise correlations can significantly affect the total information transmitted by a population of cells [67]. However, substantial noise correlations are not expected in cone isomerization rates, since in daylight these fluctuations are primarily controlled by the stochastic arrival of photons.

In treating chromatic aberration we modeled optical blur as making all cones within the scale of the blur completely redundant. In fact, the redundancy decreases with separation even within blurred regions. However, since the significant factor is the relative range of the L, M and S channel blurs, we do not expect more detailed modeling to affect our conclusions.

Like the lens of the eye, our camera lens also exhibits chromatic aberration, blurring short wavelengths more than longer ones. The effect is small, and only apparent at the highest spatial frequencies. We estimated the effect of camera blur on our results in two ways. First, we deblurred the S cone channel (adding power at high spatial frequencies) to compensate for the reduction in power at high spatial frequency due to the camera lens. Our analysis was robust to this correction. Second, we blurred the L and M cone channels (reducing power at high spatial frequencies) to match the blur in the S cone channel. Again, the results and conclusions were unchanged.

## Discussion

**Figure 10:**
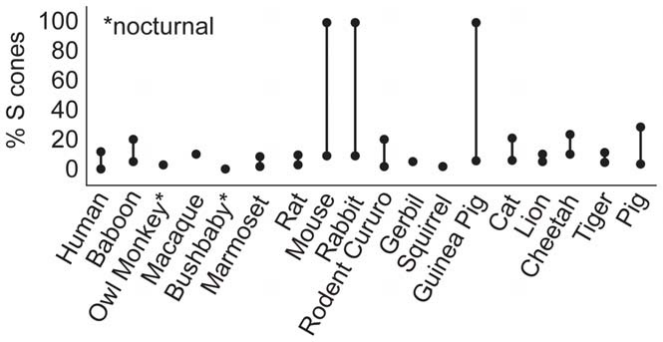
Dominance of L cones over S cones across species. Measured S cone proportion is shown for a variety of animals [2], [9], [59], [82]–[90]. For some animals, two measurements at different locations on the retina are shown. Large variation in L cone proportion indicates dorso-ventral asymmetries, like those discussed in [90].

Many mammals exhibit a significant excess of L cones over S cones (**Fig. 10**). Meanwhile, humans exhibit, on average, only a small excess of L cones over M cones [1] but the relative proportion of L and M cones varies significantly between individuals [12]. There is also topographic variation in cone proportions within a mosaic – e.g., in human retina the proportion of S cones in the central retina exceeds the proportion of S cones in the peripheral retina [1]. All these basic facts about the design of the photoreceptor mosaic seem to be explained by a single hypothesis – given the filtering properties of the eye and the optics of chromatic aberration, the overall mosaic arrangement combines with lens accommodation to maximize information transmitted from natural scenes.

Any optimization argument of this kind must hold fixed some characteristics of the system while varying others. We held fixed the number of cone types, their spectral sensitivities and the absorption of the ocular media and tested how varying cone proportions and lens accommodation wavelength changed the amount of information conveyed by the array. We treated these factors as variable because we were seeking underlying rationale for the observed cone proportions and because accommodation wavelength is under behavioral control. We could have instead varied the absorption of the ocular media or the number of cone types while holding the other factors fixed. This type of analysis will be interesting for deriving the predictions of the theory for other species.

As in the present work, we expect that in most vertebrate eyes a larger fraction of the light in natural scenes to which S cones are most sensitive never reaches the photoreceptor layer, giving an small advantage to long wavelength cones. This is because ocular media (cornea, aqueous humor, lens and vitreous) filter out more short wavelength light than long wavelength light [54],[68]. Humans, lower primates, and diurnal sciurids (squirrels), have an additional macular pigment that filters out even more short wavelength light to protect the retina from UV radiation [26]–[28], [69]–[71]. Of course, if natural scenes had much more power at short wavelengths, S cones would still have an advantage despite attenuation in the optical media. Thus, the disadvantage for S cones is the combined result of similar power at short and long wavelengths, and selective attenuation.

The present analysis offers a unified explanation for why S cones are rare, why they increase toward the periphery, and why a large variation in L/M ratio can be tolerated. A useful way to consider these results is to imagine how one might “build” a retina, cone-by cone, with the goal of transmitting as much information as possible. First consider the case where only L and S cones are available. Since the signals S cones receive are smaller, L cones are individually more valuable. Consequently, a builder would begin by using only L cones. However, as the array of L cones becomes large, each additional L cone adds progressively less value because its signals become increasingly redundant with its neighbors. Eventually the value of an additional L cone decreases sufficiently so that adding an S cone becomes advantageous, despite its smaller relative information capacity. The end result is an optimal array with mostly L cones, and a few S cones. When optical blur is included in the analysis, the redundancy in S cone signals is increased relative to the redundancy in L cone signals, making S cones even less valuable. Thus L cones dominate the array.

For L and M cones the situation is different. Since these two cone types carry similar amounts of information, adding an M cone instead of an L cone becomes advantageous much sooner, and the optimal array is more evenly mixed. Furthermore, optical blur affects L and M cone signals similarly because their spectral sensitivities are similar. The blur renders L and M cone signals - already quite redundant - even more redundant. The result is that, within the spatial extent of the blur, L and M cones have roughly equivalent value. Consequently, the information transmitted by the array changes little over a wide range of L/M proportions, although plausible variations in the parameters can give a small advantage to L or M cones. The latter might explain why, despite large variations between individuals, the human eye has, on average, more L cones, while the baboon eye has more M cones [9].

Our findings fit with a growing body of evidence that the retina allocates limited resources to maximize the information transmitted from natural scenes, subject to biophysical constraints. For example, this principle seems to explain the excess of OFF ganglion cells in the retina [72], the overlap of ganglion cell receptive fields [58], cone density distribution [73], the distribution of information traffic in the optic nerve [74], and the distribution of its axon calibers [75] (see the review [76]). Snyder, Stavenga, & Laughlin [33] pioneered this approach to understanding the design of photoreceptor arrays as maximizing information under various constraints. Their effort considered trade-offs between spatial acuity and contrast sensitivity given white noise stimuli at different intensities, but did not consider the chromatic organization of natural scenes. In a related approach to analyzing the photoreceptor array, Bayesian decision theory was used to investigate tradeoffs between monochromatic and dichromatic vision [77].

The present findings also extend a large body of work on the evolution of wavelength sensitivity. There are many examples where the peak sensitivity of a photopigment matches the most prevalent wavelength in the environment (e.g., cones of fish in Lake Baikal, [78]). There are also examples where the behavioral niches of organisms seem to influence their photoreceptor sensitivities (e.g., UV receptors in insects and birds for seeing flower patterns, and the UV receptor of falcons which detects vole urine trails that fluoresce in the ultraviolet [79]. A number of authors have, for various species, considered the optimal choice of cone opsin spectral sensitivity [21]–[24],[80],[81]. For primates, it has been suggested that trichromacy evolved to assist detection of ripe fruit on a green background [21]. It has been further argued that the spectral sensitivities of the three cone types in human might maximize information transmission from natural scenes under the constraints of chromatic aberration, diffraction, and input noise particularly in dim light [25]. These arguments suggest that the molecular properties of the cone opsin are shaped to maximize the information they transmit about behaviorally relevant stimuli, and here we find the same for the structure of the photoreceptor array.

## Materials & Methods

### Alternative formulation for single cone information

**Figure 11:**
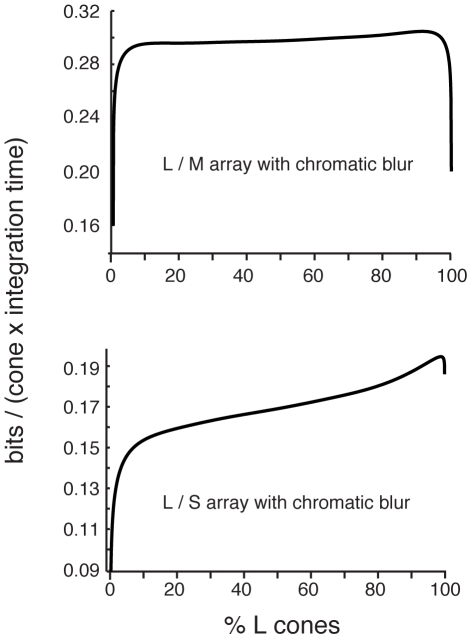
Optimal mosaic using photon noise binned calculation of single cone information (see text). Results are very similar to those in Fig. 5, using a Gaussian channel approximation. (Top) Information represented by a mixed LM array as a function of the percentage of L cones in the array. (Bottom) Information represented by a mixed LS array.

Our main results can be derived using an alternative estimate of the single cone information that does not make use of the Gaussian channel approximation, and instead directly applies Shannon's formula to the individual cone isomerization distributions. The cone signal distributions are discretized into bins with boundaries placed 2 noise standard deviations from each bin's center. This standard deviation was determined by assuming Poisson photon noise, for signals with mean intensity equal to the intensity at the center of each bin. Fig. 11 is a plot of the main result from our paper, using this method for calculating the single cone entropies. The results are qualitatively and quantitatively very similar. Note that the precise values of the cone SNRs were shown in Results to have little influence on the on the optimal cone proportions.

### Image database

Our natural image database consists of images taken in a variety of environments and lighting conditions. Most are daylight images from dry-season Botswana, but we also have images of Botswana at other times of year, Philadelphia, and locations in Southern India. Within this set we have collected low light intensity (dusk / dawn) images, close-ups, and images of the horizon with split sky/ground. The diversity of the images allows us to investigate how different color environments, behavioral needs, and activity periods (nocturnal vs. diurnal vs. crepuscular) affect the demands on spatial-chromatic information processing.

### Camera properties

Images were acquired with a Nikon D70 digital camera writing to “RAW” format. This format gives approximately 9.5 bits per pixel for each color channel (see http://www.majid.info/mylos/weblog/2004/05/02-1.html). Images were collected on a 2014×3040 photocell array with interleaved red, green, and blue sensors, then interpolated (using nearest-neighbor interpolation within each sensor class) to estimate the full red, green, and blue camera response at each pixel location. Following this interpolation, each image was downsampled by a factor of 2 to minimize aliasing artifacts from the interleaved red, green, and blue sensor sampling of the camera. At the down-sampled resolution (1007×1520), the camera resolution was 46 pixels per degree of visual angle. In our analyses we used scale invariance of natural scenes to regard the pixels as having the separation of foveal cones viewing the same scenes from a greater distance. Additional detail follows.

#### Raw image format

The D70 allows storage of images in a number of different formats. Nikon Electronic Format (NEF), records “raw” sensor values. This is a proprietary Nikon format, but its parameters are publicly available. NEF images store 6.1 megapixel 12 bit data from the image sensor as an approximately 5.00MB file. In addition, public domain software, dcraw (www.cybercom.net/~dcoffin/dcraw/), is available to read NEF images and convert them to Portable Pixel Map (PPM) format images. We used dcraw to convert the image data from NEF to PPM format, which is readable by MATLAB (The Mathworks, Natick, MA). Dcraw offers a number of options for the output files. We used it to extract the image in documentm (no color interpolation) by using the -d flag, and to write 48-bpp (48 bits per pixel, 16 bits per color channel) PPM file by using the -4 flag.

#### Geometric information

The D70's sensor provides a resolution of 2014 (v)×3040 (h) pixels. The angular resolution of the camera was established by acquiring an image of a meter stick from a distance of 123 cm. The corresponding angular resolution is 92 pixels per degree both horizontally and vertically.

#### Mosaic pattern

The D70 employs a mosaiced photosensor array to provide RGB color images. That is, each pixel in a raw image corresponds either to an R, G, or B sensor. R, G, and B values can then be interpolated to each pixel location. Raw mosaic sensory values extracted by dcraw were used for our various calibration measurements described below. The mosaic pattern of the D70 camera, starting in the upper left corner of the image, is
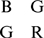
This sub-mosaic pattern then tiles the rest of the full 2014×3040 image.

#### Dark subtraction

Digital cameras typically respond with positive sensor values even when there is no light input (i.e. when an image is acquired with an opaque lens cap in place.) This dark response can vary between color channels and with exposure duration. We measured the dark response of each color channel as function of exposure duration. This was done by placing a dark lens cap on the lens, setting the aperture to its minimum (f22) and acquiring data in a room with the lights turned off. The dark response values were subtracted from actual response values as part of our image preprocessing.

#### Image quantization

Although the D70 has native 12-bit per pixel intensity resolution, the D70 appears not to write the raw 12-bit data to the NEF file. Rather, some quantization/compression algorithm is applied which converts the image data from 12 bit intensity resolution to approximately 9.5 bit resolution (www.majid.info/mylos/weblog/2004/05/02-1.html). Dcraw corrects for any pixel-wise nonlinearity introduced by this processing (see description of verification below), but it cannot, of course, recover the full 12-bit resolution. The pixel values in the file extracted by dcraw range between 0 and 16400. These are the values we analyze in all that follows. We do not have a direct estimate of the true intensity precision of this representation.

#### Response linearity

Fundamental to digital camera calibration is a full description of how image values obtained from the camera relate to the intensity of the light incident the sensors. Two common properties of digital camera sensor responses can complicate calibration. First, the camera response function may be nonlinear, so that a doubling of the input intensity does not result in a doubling of the sensor response. Second, camera responses are quantized, rather than varying continuously with the intensity of the light input. We characterized the D70's response as a function of light intensity.

To measure the linearity of the camera response, we measured the light reflected from a white reflectance standard at each of 55 exposure durations, 1/8000 *s* to 30 *s*. The aperture was held constant at f11. Response values were obtained by extracting and averaging RGB values from a 60×60 pixel region that contained image values from standard.

The intensity values corresponding to the fastest exposure durations were not substantially different from the dark response values. Also, the camera saturated in at least one channel for exposure durations greater than 1/3 s. Therefore, data were analyzed only for exposure durations longer than 1/2000 s, and shorter than 1/3 s. The data deviated from linearity for shorter exposure durations. The linear range, expressed in terms of camera response values is from 100 sensor units to the saturation value of the sensors, 16400 sensory units. These data did not distinguish two possible causes of the deviation from linearity. This effect may have been due to changes in the camera's shutter precision at short exposure durations or there may have been be a nonlinearity in the camera sensor response at low response levels.

To distinguish these two possibilities, we made additional measurements with camera duration and aperture held fixed at 1/100 s and f14, respectively. We varied the light intensity by placing neutral density filters between the light source (projector) and the white standard. The camera sensors were linear at high light levels (below saturation) but clearly nonlinear at low light levels (sensor response less than 100 units.) These data suggest that the nonlinearity is in the camera sensor response at low response levels. The camera's shutter precision appears to operate properly at exposure durations at least as short as 1/100 s.

#### Aperture test

If a camera's aperture is operating properly, the aperture size (f-number) expresses the relative amount of light reaching the photosensors, per unit area. For the same light source, the intensity per unit area for f-number x, I(x), relative to the intensity per unit area for f-number y, I(y) is given by: I(x) = (x/y)^2^ I(y). We tested aperture performance by measuring the sensor response as a function of exposure duration for all color channels, at three different aperture sizes (f5, f11, and f16). Applying the above relation, we compared the response function at f5 to the response functions at f11 and f16 transformed to their equivalent f5 values. After the correction is made, the sensor response as a function of exposure duration in each color channel for aperture sizes f11 and f16 should be coincident with the response function for the corresponding color channel at aperture size f5. The response functions were coincident after correction. As a convergent measure of aperture reliability, we directly measured the sensor response to a fixed source image and exposure duration at various aperture sizes (23 aperture sizes between f1.8 and f22) and confirmed that the response was proportional to the square of the f-number.

#### Spectral response

We measured the spectral sensitivities of the camera sensors. A slide projector, the Nikon D70 Camera, and a spectroradiometer were positioned in front of the white test standard. Light from the projector was passed through one of 31 narrowband monochromatic filters, with peak transmittances at 10 nm intervals between 400 and 700 nm. For each filter, a digital picture (f-number held at 1.8 and varying exposure duration) and a spectroradiometer reading were taken. This process was repeated, once with ascending wavelengths and once with descending wavelengths. At each wavelength, The R,G,B data was dark subtracted and inversely scaled by the exposure duration and the total light power measured by the spetroradiometer to yield the spectral sensitivity at that wavelength.

The measured spectral sensitivities were used to generate predictions of the camera sensor response to arbitrary light sources. To check the accuracy of our model, we compared predicted and measured response values for the 24 swatches of the Macbeth color checker. For each swatch, we took a spectroradiometer reading and a digital photo. The sensor RGB responses were well predicted by the measured camera spectral sensitivities and a measurement of the light spectrum incident at the camera.

#### Predicted L, M, and S cone response

A linear mapping derived from our measured camera spectral sensitivities and the Stockman & Sharpe (2000) cone spectral sensitivities was used to transform signals represented in RGB image space into equivalent L, M, and S cone coordinates. In making this transformation, we took the aperture and exposure duration for each image into account. To the extent that L, M, and S cone signals are not a linear transformation of R, G, and B camera signals, the linear transform will have some error, including the possible prediction of negative cone isomerization rates. This could occur when pixel values are near zero or when one color channel is much higher than the others (e.g., R is high, but G and B are near zero). However, because we used bright daylight images and because color channels in natural images are highly correlated, negative LMS values were not observed in our images.

The LMS coordinates were scaled to yield estimates of photoisomerization rates for each cone class (R* s^−1^). The scaling factors were estimated by computing cone isomerization rates from measured spectra using the procedures described by Yin, Smith, Sterling, & Brainard [37] for guinea pig, but substituting appropriate parameters for human foveal cones (human peak photopigment sensitivities, optical densities, photoreceptor dimensions, ocular media transmittance). These were then regressed separately for each cone class against the corresponding cone coordinate to yield the conversion factor for that cone class.

#### Camera chromatic Modulation Transfer Function

We measured the spatial Modulation Transfer Function (MTF) for the R, G, and B color channels. To do so, we imaged a high contrast black and white grating at a series of camera distances and computed the image contrast at the corresponding spatial frequencies. The MTF was similar for the R and G channels, but more severe for the B channel. We used the data to estimate the spatial MTF of the L, M, and S cone image planes and as described in the text verified that the different MTF of the camera B channel did not influence our results.

### Optical point spread function calculations

Point spread functions (PSFs) were estimated from wavefront aberration measurements obtained for 13 subjects, reported by Chen, et al. [14]. For each subject, the wavefront aberration measurements characterize individual observer optical aberrations and allow calculation of the PSF for various choices stimulus wavelength, pupil diameter, and accommodative state. All of our calculations were performed for a 3mm pupil. The code to convert wavefront aberration measurements to polychromatic PSF as a function of accommodation was kindly provided to us by Heidi Hofer, along with the tabulated wavefront aberration measurements required for the calculations.

We were interested in how the PSF seen by the L cones traded off the PSF seen by the S cones as accommodation varied. For each observer, we computed the PSF for each stimulus wavelength (372 nm to 700 nm at 4 nm steps) for a range of accommodative states (nominal wavelengths of accommodation 372 nm to 700 nm at 4 nm steps). For each observer and accommodative state, we then obtained the PSF seen by each cone class by weighting the individual wavelength accommodative states by the spectral sensitivity of that cone class. This provided individual observer data on how the PSFs seen by the two cone classes varied with accommodation. To combine data for each observer, we found for each observer the accommodative state that minimized various weighted sums of the widths of the PSFs seen by the L and S cones. PSF width for this optimization was obtained from the circular average of each computed PSF. For each choice of L and S cone weights in the minimization, the optimized PSFs for each cone class were circularly averaged within each observer and then averaged across observers. The result of this analysis is shown in **Fig. 6**, where the computed points trace out the desired tradeoff, with widths obtained from the final average across observer PSFs for each choice of S and L cone weights. The endpoints of the curve shown provide the minimum width PSF obtainable for each of two cone classes, when no weight was attached to the width of the PSF for the other class. It was important to separately optimize the accommodative state for each observer for each weight choice, because for any particular weight choice different observers' PSFs were optimized by different nominal wavelengths of accommodation.

The smooth fit to the PSF tradeoff shown in **Fig. 6** was used to resample the tradeoff frontier for the calculations shown in **Fig. 7**. We also used the general procedure described above to find the PSFs seen by the L, M and S cones when the accommodative state was set to minimize the average width of the L and M cone PSFs.
